# Life Expectancy and Death by Diseases of the Circulatory System in Patients with Bipolar Disorder or Schizophrenia in the Nordic Countries

**DOI:** 10.1371/journal.pone.0067133

**Published:** 2013-06-24

**Authors:** Thomas Munk Laursen, Kristian Wahlbeck, Jonas Hällgren, Jeanette Westman, Urban Ösby, Hassan Alinaghizadeh, Mika Gissler, Merete Nordentoft

**Affiliations:** 1 National Centre for Register-based Research, Aarhus University, Aarhus, Denmark; 2 Psychiatric Centre Copenhagen, Faculty of Health Sciences, University of Copenhagen, Copenhagen, Denmark; 3 Nordic Research Academy in Mental Health, Nordic School of Public Health, Gothenburg, Sweden; 4 Department of Medical Epidemiology and Biostatistics (MEB), Karolinska Institutet, Stockholm, Sweden; 5 Centre for Family Medicine, Department of Neurobiology, Care Sciences, and Society, Karolinska Institutet, Stockholm, Sweden; 6 Department of Molecular Medicine and Surgery, Neurogenetics, Karolinska Institutet, Stockholm, Sweden; 7 Center for Molecular Medicine, Karolinska Institutet, Stockholm, Sweden; 8 Department of Psychiatry, Tiohundra AB, Norrtälje, Sweden; 9 THL National Institute for Health and Welfare, Helsinki, Finland; Catholic University of Sacred Heart of Rome, Italy

## Abstract

**Objective:**

Excess mortality from diseases and medical conditions (natural death) in persons with psychiatric disorders has been extensively reported. Even in the Nordic countries with well-developed welfare systems, register based studies find evidence of an excess mortality. In recent years, cardiac mortality and death by diseases of the circulatory system has seen a decline in all the Nordic countries, but a recent paper indicates that women and men in Denmark, Finland, and Sweden, who had been hospitalised for a psychotic disorder, had a two to three-fold increased risk of dying from a cardiovascular disease. The aim of this study was to compare the mortality by diseases of the circulatory system among patients with bipolar disorder or schizophrenia in the three Nordic countries Denmark, Sweden, and Finland. Furthermore, the aim was to examine and compare life expectancy among these patients. Cause specific Standardized Mortality Rates (SMRs) were calculated for each specific subgroup of mortality. Life expectancy was calculated using Wiesler’s method.

**Results:**

The SMR for bipolar disorder for diseases of the circulatory system was approximately 2 in all countries and both sexes. SMR was slightly higher for people with schizophrenia for both genders and in all countries, except for men in Denmark. Overall life expectancy was much lower among persons with bipolar disorder or schizophrenia, with life expectancy being from 11 to 20 years shorter.

**Conclusion:**

Our data show that persons in the Nordic countries with schizophrenia or bipolar disorder have a substantially reduced life expectancy. An evaluation of the reasons for these increased mortality rates should be prioritized when planning healthcare in the coming years.

## Introduction

Excess mortality from diseases and medical conditions in persons with psychiatric disorders has been extensively reported [1]. Even in the Nordic countries with well-developed welfare systems, register based studies find continued evidence of an excess mortality [2]–[7]. In the Nordic countries, women and men with severe mental disorders have an approximately 15 and 20 years shorter life expectancy, respectively, than the general population [8]. Patients with bipolar disorder or schizophrenia seem to be a particular vulnerable patient group [6].

Diseases of the circulatory system are the most common cause of death in the general population in Western countries [9], [10], including Denmark, Finland, and Sweden. In recent years, cardiac mortality has seen a decline in all the Nordic countries, but a recent paper indicates that women and men in Denmark, Finland, and Sweden, who had been hospitalised for a psychotic disorder, had a two to three-fold increased risk of dying from a cardiovascular disease [11]. A study from Denmark showed that patients with bipolar affective disorder or schizophrenia still have an overall cardiac mortality that is higher than in the general population, and that the excess mortality tends to be even larger in the most recent calendar time period [12].

The aim of this study was to compare the mortality by diseases of the circulatory system among patients with bipolar disorder or schizophrenia in the three Nordic countries Denmark, Sweden, and Finland. Furthermore, the aim was to examine and compare life expectancy among these patients.

## Methods

### Study Population and Follow-up Period

The Nordic countries have a long tradition of highly comprehensive population registers, which are based on individual data. Every resident in the Nordic countries has their own unique personal identification number which enables linkage of data between registers. The universal population register coverage makes it possible to follow entire populations in the three countries with a combined total population of 20 million people. For this study, the population at risk consisted of all persons with a hospitalization with a main diagnosis of bipolar disorder or schizophrenia to a psychiatric ward in Denmark, Finland, or Sweden. Information on psychiatric hospitalization was extracted from population based discharge registers.

The follow-up for the population at risk began on 1 January 2000 or on the cohort member’s 15th birthday, whichever came last. The follow-up ended on 1 January 2007, or at the day of death, whichever came first. We choose the year 2000 as the starting point as we did not want to include information from a period with a much shorter life expectancy in the Nordic population.

### Assessment of Psychiatric Illness and Cause of Death

Patients included were diagnosed with schizophrenia or bipolar disorder. Thus, individuals diagnosed with schizoaffective disorder were not included, neither in the schizophrenia nor the bipolar disorder diagnostic groups.

#### Denmark

Data on psychiatric hospitalizations of the entire Danish population were drawn from the Danish Central Psychiatric Case Register [13]. The register includes data on all psychiatric inpatient admissions in Denmark since April 1, 1969. Only patients admitted to a psychiatric ward were included. In Denmark, the diagnostic system used until December 31, 1993 was ICD-8 [14], and from January 1, 1994, ICD-10 [15]. We identified all persons with schizophrenia (F20/295 excluding 295.49 and 295.79) or bipolar affective disorder (F30, F31/296.19, 296.39). The Danish Cause of Death Register [16] contains computerized information about all deaths of Danish citizens and residents, including date and causes of death. The validity of the register is high [16].

#### Finland

Finnish data on psychiatric hospitalization was drawn from The Finnish Hospital Discharge Register (FHDR) which includes information on all inpatient hospitalization since 1969. The ICD-9 classification was used during the period 1987–1995 and the ICD-10 from 1996 onwards. We identified all persons with schizophrenia (F20/295 excluding 295.4 and 295.7) or bipolar affective disorder (F30, F31/296.2 to 296.7). The validity of the FHDR has been found to be of good quality [17]. The Cause of Death Register in Finland has information on all causes of death for persons living in Finland. The completeness of the register is excellent since all diagnosis routinely passes a validation by Statistics Finland [18].

#### Sweden

The Swedish Hospital Discharge Register covers all hospital admissions and discharges in all regions in Sweden since 1987. The register includes data since 1964, but some regions are not included for years 1964–1986. The ICD-9 diagnostic system was used during the period 1987–1996 and ICD-10 from 1997 onwards. We identified all persons with schizophrenia (F20/295 excluding F, H) or bipolar affective disorder (F30, F31/296 excluding B, W, and X). The Swedish register has a high level of completeness [19]. The Swedish Cause of Death Register contains information about all deaths in Sweden and the coding error is estimated from a sample of deaths and was only 3% [20].

In all three countries persons were categorized as having bipolar disorder/schizophrenia from the date of their admission to a psychiatric hospital with such a diagnosis. Patients with a diagnosis of both schizophrenia and bipolar disorder were categorized as having schizophrenia from the day of first diagnosis of schizophrenia. Because data in Sweden was not complete before 1987, we only used information on schizophrenia and bipolar disorder from 1987 and onwards in all countries. We thus had a 13 year period from 1987 to 1999 to find persons with a schizophrenia/bipolar disorder before start of follow-up. All persons with a first contact of schizophrenia/bipolar disorder in 2000 or thereafter were included at the day of first contact with a diagnosis. E.g. if a person had his/her first schizophrenia or bipolar diagnosis on July 31, 2004 the person was not in the population at risk from January 1, 2000 to July 31, 2004; but was in the population at risk from August 1, 2004 onwards.

The underlying cause of death was used in all the three Cause of Death Registers. Over-all mortality was examined: ICD-10 A00-Y98, deaths from diseases and medical conditions ICD 10: A00-R99, and deaths from external causes ICD-10: V01-Y989. Cause-specific death of Chapter IX: Diseases of the circulatory system (Chapter I), was defined as ischaemic heart disease (I20–25), cerebrovascular disorder (I60–69) and a miscellaneous group of Chapter I (I00-I20, I26-I59, I70-I99).

### Statistical Analyses

In the cohort study, standardized mortality ratios (SMR) was calculated, comparing the mortality rates among persons with bipolar disorder or schizophrenia with the rate in the general population [21]. In order to make a reliable comparison of SMRs between the countries the crude mortality rates were age-standardized by using the Nordic standard population in 2000 [22]. I.e. for each 5-year group we had the percentage of the population in that age group. We then insured that the population we examined had the same age distribution as the Nordic standard population in all three countries. That is, we standardized the SMR in each of the three countries to the same age distribution to ensure that the differences in SMR was not merely due to differences in the age distribution in the three countries, see chapter 3 in Rothman et al. [23]. If no age standardization is performed it is difficult to compare SMRs. If, as an example the Swedish population is much older than the Danish population, it would appear that the persons in Sweden die at a higher rate, simply because the mortality rate is higher in older person. When we standardize we only compare persons with the same age to one another. Note that the 5 year age group was only used in the standardization of the SMRs. Cause specific SMRs were calculated for each specific subgroup of mortality in the same way. We calculated 95% confidence intervals using the approximation described in the book by Breslow and Day [24].

For persons with schizophrenia or bipolar disorder, we calculated life expectancy at 15 years by using Wiesler's method with one-year age stratification [25], [26]. Life expectancy at a given age represents the average number of years of life remaining if a group of persons at that age were to experience the mortality rates for a particular year over the course of their remaining life. Life expectancy at 15 years is a summary measure of the age specific all-cause mortality rates in an area in a given period [25], [26]. The life expectancy among these persons was compared to that of the general population, and the differences in life expectancies were calculated. The probability of getting a diagnosis of schizophrenia or bipolar disorder before the age of 15 is virtually zero in the Nordic countries, and thus the reason for starting follow-up at that time point in life. If a person died half way through the follow-up period, they only contributed half the period to the time at risk.

The number of deaths that would not occur if the group of persons with a psychiatric disorder had the same mortality as the general population were calculated for each subgroup of psychiatric disorder, sex and country by the formula: N*((SMR-1)/SMR), where N is the number of deaths and SMR is the SMR of the excess mortality [27].

### Ethics

All personal identification numbers were anonymized before the analysis of data began. The study was approved by ethical committees and data protection agencies in each of the three countries. According to the legislation in the three countries, no informed consent from participants was needed because data were analyzed anonymously.

The study was approved by the Danish Data Protection Agency (2000-41-0307).

The permission to create the Finnish research database was given by STAKES (National Research and Development Centre for Welfare and Health, currently THL National Institute for Health and Welfare), Statistics Finland and National Social Insurance Institution. The Data Protection Ombudsman (Tietosuojavaltuutettu) gave his statement before the study data was created, as requested by the national legislation on data protection.

We have received ethical permission for this study from the ethics committee in Gothenburg, Sweden (Dnr 130-08).

## Results

By the end of 2006, the total population in Denmark, Finland, and Sweden included 16.3 million inhabitants aged 15 years or more. A total patient population of 39,375 (bipolar affective disorder) and 66,088 (schizophrenia) persons was under risk of dying in the period 2000–2006, respectively. Of them 5,436 (bipolar disorder) and 9,535 (schizophrenia) did die during the seven year follow-up, Table 1 and 2.

**Table 1:** Standardized mortality rates (SMR) for overall mortality, natural/unnatural causes and a sub-division of diseases of the circulatory system (DCS; ICD I00-I99) for men with bipolar disorder/schizophrenia compared to the general population in Denmark, Finland, and Sweden with 95% confidence intervals.

MEN	Denmark	N*	Finland	N*	Sweden	N*
**Bipolar disorder**						
**Total risk population**	4280		4489		7367	
All causes of death	2.2 (2.0–2.3)	770	2.8 (2.5–3.0)	485	2.0 (1.9–2.2)	1097
Natural deaths**	1.9 (1.7–2.0)	635	2.1 (1.9–2.4)	278	1.8 (1.6–1.9)	883
All external causes of death	7.8 (6.6–9.3)	135	8.5 (7.4–9.7)	207	6.3 (5.5–7.2)	214
Overall DCS (I00-I99)	2.0 (1.7–2.2)	222	2.2 (1.8–2.6)	128	1.7 (1.5–1.9)	408
Ischaemic heart disease (I20-I25)	1.9 (1.5–2.3)	94	2.0 (1.6–2.6)	74	1.6 (1.4–1.8)	200
Acute myocardial infarc. (I21)	1.9 (1.4–2.6)	44	1.9 (1.3–2.7)	30	1.7 (1.4–2.1)	124
Cerebrovascular disease (I60-I69)	2.0 (1.5–2.6)	53	1.4 (0.7–2.5)	11	1.6 (1.3–2.0)	78
Other DCS	2.0 (1.6–2.5)	75	3.5 (2.5–4.7)	43	2.0 (1.7–2.4)	130
**Schizophrenia**						
**Total risk population**	12006		10369		14307	
All causes of death	3.0 (2.8–3.1)	1515	3.3 (3.2–3.5)	1439	2.9 (2.8–3.0)	2126
Natural deaths**	2.7 (2.6–2.9)	1169	3.2 (3.0–3.4)	1108	2.6 (2.5–2.8)	1754
All external causes of death	7.4 (6.6–8.2)	346	4.8 (4.3–5.4)	331	6.6 (6.0–7.4)	372
Overall DCS	1.8 (1.6–2.1)	294	2.9 (2.6–3.1)	471	2.5 (2.3–2.7)	783
Ischaemic heart disease (I20-I25)	1.6 (1.3–1.9)	125	2.5 (2.2–2.8)	263	2.5 (2.3–2.8)	439
Acute myocardial infarc. (I21)	1.6 (1.2–2.1)	64	2.5 (2.1–3.0)	121	2.3 (2.0–2.6)	238
Cerebrovascular disease (I60-I69)	1.6 (1.2–2.1)	54	3.2 (2.6–4.0)	78	2.4 (2.0–2.8)	124
Other DCS	2.4 (2.0–2.9)	115	3.8 (3.2–4.5)	130	2.6 (2.3–3.0)	220

Subdivided by causes of death.

* Number of deaths.

** All diseases and medical conditions.

**Table 2:** Standardized mortality rates (SMR) for overall mortality, natural/unnatural causes and a sub-division of diseases of the circulatory system (DCS; ICD I00-I99) for women with bipolar disorder/schizophrenia compared to the general population in Denmark, Finland, and Sweden with 95% confidence intervals.

WOMEN	Denmark	N*	Finland	N*	Sweden	N*
**Bipolar disorder**						
**Total risk population**	6 821		5 430		10988	
All causes of death	2.0 (1.9–2.1)	1098	2.5 (2.2–2.8)	382	2.3 (2.2–2.4)	1604
Natural deaths**	1.8 (1.7–1.9)	982	2.0 (1.8–2.3)	271	2.0 (1.9–2.1)	1396
All external causes of death	7.0 (5.8–8.3)	116	10.3 (8.4–12.4)	111	9.0 (7.8–10.3)	208
Overall DCS (I00-I99)	1.7 (1.5–1.9)	308	2.1 (1.7–2.4)	121	1.8 (1.7–1.9)	566
Ischaemic heart disease (I20-I25)	1.6 (1.3–1.9)	112	1.8 (0.8–3.6)	8	1.9 (1.7–2.2)	255
Acute myocardial infarc. (I21)	1.7 (1.2–2.2)	52	1.6 (1.0–2.3)	24	2.2 (1.9–2.6)	153
Cerebrovascular disease (I60-I69)	1.6 (1.3–2.0)	84	2.2 (1.5–3.1)	33	1.8 (1.6–2.2)	149
Other DCS	1.8 (1.5–2.1)	112	2.5 (1.7–3.6)	30	1.6 (1.4–1.9)	162
**Schizophrenia**						
**Total risk population**	8 424		10 466		10516	
All causes of death	2.7 (2.5–2.8)	1014	3.9 (3.7–4.1)	1624	2.9 (2.8–3.0)	1817
Natural deaths**	2.4 (2.3–2.6)	864	3.0 (2.8–3.2)	1141	2.6 (2.5–2.8)	1647
All external causes of death	8.9 (7.6–10.5)	150	8.0 (6.8–9.2)	483	8.9 (7.6–10.4)	170
Overall DCS (I00-I99)	2.3 (2.0–2.6)	237	2.7 (2.5–3.0)	472	2.6 (2.4–2.7)	701
Ischaemic heart disease (I20-I25)	2.2 (1.8–2.7)	90	2.6 (2.3–3.0)	248	2.7 (2.4–3.0)	307
Acute myocardial infarc. (I21)	2.3 (1.7–3.0)	49	2.7 (2.2–3.2)	117	2.2 (1.9–2.5)	169
Cerebrovascular disease (I60-I69)	1.9 (1.4–2.4)	54	2.3 (1.9–2.8)	106	2.2 (1.9–2.6)	156
Other DCS	2.9 (2.3–3.6)	93	3.7 (3.0–4.4)	118	2.7 (2.4–3.0)	238

Subdivided by causes of death.

* Number of deaths.

** All diseases and medical conditions.

All-cause mortality was high among persons with bipolar disorder, resulting in a SMR for men in Finland equaling 2.8, in Denmark 2.2, and in Sweden 2.0. The SMR for women was 2.5 in Finland, 2.3 in Sweden and 2.0 in Denmark. The SMRs for persons with schizophrenia, was even higher with a SMR for men equaling 3.3, 3.0 and 2.9 in Finland, Denmark, and Sweden, respectively, Table 1 and 2. For women with schizophrenia the SMR was 3.9, 2.7 and 2.9 in Finland, Denmark, and Sweden, respectively.

In general SMR for mortality from external causes (accidents, suicide, and homicide) was very high (range from 4 to 10). In Finland, SMR for death from external causes was in general higher in persons with bipolar disorder than in persons with schizophrenia, Table 1 and 2.

The SMR for bipolar disorder for different subgroups of diseases of the circulatory system (DCS) was approximately 2 in all countries and both sexes, and thus lower than the all-cause SMRs, Table 1and 2. SMR for DCS among persons with schizophrenia was also lower than overall SMRs in Denmark and Finland, but not in Sweden. Subdivision of DCS in ischaemic heart disease (characterized by reduced blood supply to the heart), cerebrovascular disease (characterized by brain dysfunctions related to disease of the blood vessels), and a miscellaneous group (e.g. rheumatic heart diseases, hypertensive diseases, diseases of arteries/veins) reviled no large variation of SMRs in Denmark and Sweden, but SMRs were higher in the miscellaneous group in Finland. The same pattern was seen among persons with schizophrenia.

Life expectancy in general is higher in Sweden than in Denmark and Finland. Similarly, life expectancy among persons with bipolar disorder and schizophrenia was higher in Sweden than in the other two Nordic countries. Overall life expectancy was much lower among persons with bipolar disorder or schizophrenia, with life expectancy being 11 to 20 years shorter. Persons with bipolar disorder in Denmark and Sweden had higher life expectancy than persons with schizophrenia. This was in contrast to Finland where persons with bipolar disorder had shorter life expectancy, Table 3.

**Table 3:** Remaining life expectancy at ***age 15*** and difference in life expectancy compared to general population among patients with bipolar disorder and schizophrenia in Denmark, Finland, and Sweden.

	Denmark		Finland			Sweden	
MEN	Life expectancy	Difference*	Life expectancy		Difference*	Life expectancy	Difference*
General population	60.7	–	60.7		–	63.2	–
Bipolar disorder	47.1	13.6	40.9		19.8	50.5	12.7
Schizophrenia	40.7	20.0	43.6		17.1	44.3	18.9
**WOMEN**	**Life expectancy**	**Difference** *	**Life expectancy**	**Difference** *		**Life expectancy**	**Difference** *
General population	65.3	–	67.5	–		67.6	–
Bipolar disorder	54.3	11.0	51.3	16.2		55.0	12.6
Schizophrenia	48.8	16.5	51.9	15.6		50.7	16.9

Wiesler's method.

* Compared to the general population.

During the years 2000 to 2006 the number of deaths in all three countries that would not have occurred in the hypothetical situation that the individuals with a psychiatric disorder had the same mortality as the general population was 2,960 (out of 5,436 deaths, 54%) in the group of persons with bipolar disorder and 6,424 (out of 9,535 deaths, 67%) in the group of schizophrenic persons. The number of deaths in all three countries that would not have occurred if persons with bipolar disorder or schizophrenia had not had an excess mortality from I00-I99 was 781 and 1,770, respectively. If no excess mortality from external causes was present the numbers was 865 and 1,322, respectively.

## Discussion

In a study including information from 20 million individuals from three Nordic countries with complete national coverage, and with more than 100,000 persons with bipolar disorder or schizophrenia, we found that overall mortality increased 2- to 3-fold compared to the general population. Among persons with bipolar disorder or schizophrenia, life expectancy was generally 12 to 20 years reduced for men and 11 to 17 years reduced for women, compared to the general population. Persons with bipolar disorder had a slightly better prognosis than people with schizophrenia, as only men in Finland with schizophrenia had overlapping confidence intervals of the overall SMR with men with bipolar disorder. In all other cases the overall SMR for people with schizophrenia was significantly higher (no overlapping confidence intervals) than for bipolar disorder.

### Comparison between the Study Countries

Several Danish studies have shown that persons with bipolar disorder have a slightly better prognosis than persons with schizophrenia, e.g. lower overall mortality [6], higher fertility rates [28], and receive more somatic care [29]. In the beginning of the 20^th^ century, Emil Kraepelin proposed a dichotomization of psychotic illness in two separate disorders; bipolar disorder (manic depression) and schizophrenia (dementia praecox). One of the key features of his dichotomization was the better prognosis for persons with bipolar disorder compared to those with schizophrenia [30]. In this study SMRs for persons with bipolar disorder were lower compared to SMRs for schizophrenia in all three study countries. Thus, this key feature of the dichotomization still applies for today’s patients. It should however be noted that although men and women in Finland with bipolar disorder have a lower overall mortality measured by SMR than men and women with schizophrenia, the potential loss of living years are larger among persons with bipolar disorder. This seems to be a paradox, but, the explanation is that e.g. Finnish men with bipolar disorder have a very high mortality rate in the age groups between 15 and 44 years: a person who dies at a young age contributes many years to the potential loss of living years, and thus this measure becomes high although SMRs are not that high.

The SMRs had a tendency to be slightly higher in Finland. Apart from this difference the mortality rates and life expectancy are remarkably similar in the three countries. The difference may be related to problems in health care access in Finland. In Denmark and Sweden, public health care is tax-funded and highly available, but in Finland availability is compromised by a fragmented health system, where health care access is more dependent on employment status and income level. This may create higher levels of health inequity in Finland than in Denmark or Sweden.

### Comparisons with other Studies

A large number of reviews and commentaries have recently been made on the excess mortality from cardiovascular disorders in patients with schizophrenia and bipolar disorder [31]–[41]. Most studies find that cardiovascular disorder is the leading cause of death in both disorders. On average the excess mortality rate in people with schizophrenia resulted in a SMR equaling 2–3 and a SMR for persons with bipolar disorder equaling a little less than two.

The main conclusions on the reasons for the excess mortality in these reviews were: persons with schizophrenia and bipolar disorder tended to have an unhealthy lifestyle especially regarding diet, smoking, alcohol, and exercise; some antipsychotic medicine may have negative side effects, although no clear differences between different antipsychotics have been found; physical illnesses in persons with schizophrenia or bipolar disorder are apparently treated insufficiently or diagnosed too late. And lastly, the risk of suicide is very high [40].

Some aspects of the present study samples are worth noting. The schizoaffective subgroup, often included in the schizophrenia group, with a generally more favourable outcome than schizophrenia, was not included in our analyses. For the bipolar disorder sample, ICD diagnoses does not differentiate between bipolar I or bipolar II disorder. Thus, bipolar I would tend to be overestimated, since those patients to a larger extent would be identified by a hospital diagnosis compared to bipolar II patients. Also, bipolar disorder patients are much less likely to be treated with antipsychotic medication. In a recent study from Stockholm County, around 30% of persons with bipolar disorder treated at a specialized affective disorder unit medicated with antipsychotics [42], while in schizophrenia almost none would be without antipsychotics, and also taking larger doses. The difference in antipsychotic treatment might at least partially explain the difference in cardiovascular mortality outcome found in this study.

Reviews have found a huge reduction in life expectancy of approximately 10 to 20 years in persons with schizophrenia. The reductions of life expectancy among persons with bipolar disorder seem to be slightly smaller: approximately 10 years in a study from London [43], and 13 years in a Danish study made on data similar to the Danish data used in Table 3 [44]. A reduction of 11 to 20 years in life expectancy is higher than what is associated with most other risk factors for premature death such as diabetes (up to 2 years) [45], smoking (10 years) [46], and severe obesity (10 years) [47]. Thus, it is evident that action to prevent this unacceptable high mortality must be taken, as concluded by all of the above mentioned reviews and several recent comments [48], [49].

### Preventing the High Mortality

By reducing mortality in the group of persons with schizophrenia or bipolar disorder (approximately 100,000 persons) to the level of the general population, 9,384 deaths would have been avoided in the seven year period of this study in the three Nordic countries. This is a theoretical calculation, based on the combined national register data, but it shows that although schizophrenia and bipolar disorder are rare disorders, the extent of the increased mortality is a substantial public health problem that has not been addressed.

People with schizophrenia or bipolar disorder should be targets for routine health screenings and for targeted health promotion. Physical health problems needs to be better acknowledged in mental health care and integrated with psychiatric treatment. Access to good quality health care needs to be ensured for people with schizophrenia and bipolar disorder by fighting stigma and discrimination of people with mental disorders. Life expectancy of people with severe mental disorders should be introduced as a routine performance indicator of health outcome systems.

### Conclusions

In spite of recent focus on the excess mortality among persons with schizophrenia or bipolar disorder, this study show that persons with schizophrenia or bipolar disorder still have a substantially reduced life expectancy. Persons with schizophrenia and bipolar disorder constitute a vulnerable group of patients who need special attention. Even in the Nordic countries with well-developed welfare and public health systems this group of patients experiences an unacceptable high mortality. At present it is pertinent to evaluate why they experience high mortality rates and what could be done to improve their life expectancy.
